# Activation of murine 'T' lymphomas in the presence of a human myeloma cell line, RPMI-8226, in vivo.

**DOI:** 10.1038/bjc.1988.63

**Published:** 1988-03

**Authors:** B. C. Millar, J. L. Millar, A. Jones, S. W. Feary, D. Robertson, J. B. Bell

**Affiliations:** Section of Medicine, Institute of Cancer Research, Sutton, Surrey, UK.

## Abstract

**Images:**


*Br. J. Cancer* (1988), **57**, 290–292 © The Macmillan Press Ltd., 1988

SHORT COMMUNICATION

### Activation of murine 'T' lymphomas in the presence of a human myeloma cell line, RPMI-8226, *in vivo*

B.C. Millar[1], J.L. Millar[1], A. Jones[1], S.W. Feary[2], D. Robertson[3] & J.B.G. Bell[1]

[1]*Section of Medicine;* [2]*Department of Human Genetics; and* [3]*Department of Pathology, Institute of Cancer Research and Royal Marsden Hospital, Sutton, Surrey, SM2 5PX, UK*

Although athymic nude mice are an established model for the maintenance of human xenografts *in vivo* (Sharkey *et al.*, 1978), they can exhibit a high incidence of malignant lymphomas (Custer *et al.*, 1973) which may be associated with chronic antigen stimulation resulting in the activation of endogenous murine leukaemic viruses, MuLV, (Baird *et al.*, 1982). Also, xenotropic and ectopic MuLV can infect transplanted tumour cells (Hirsch *et al.*, 1972; Todaro *et al.*, 1973) and intact virions may be recovered from such tumours *in vitro* after passage in mice (Price *et al.*, 1975). These phenomena can become a serious problem in the routine handling of human tumours; either because the resulting tumour could become murine rather than human or because the addition of murine viral proteins to the complement of human cell proteins could complicate biochemical and immunological studies of the tumour as well as efforts to identify possible human retroviruses endogenous to a particular tumour.

At the Royal Marsden Hospital there is considerable interest in the biology and drug sensitivity of malignant myeloma. The human myeloma cell line, RPMI-8226, (Matsuoko *et al.*, 1967) is used routinely in our laboratory as a model for the disease and it was intended to establish these cells *in vivo* as a xenograft. Electron micrographs of the cells show that they are plasmablastoid with a well developed endoplasmic reticulum (Figure 1a). They are hyperdiploid, exhibit lambda immunoglobulin on the cell surface as well as HAN PCl, which is characteristic of plasma cells (Mertens *et al.*, 1985), and have a doubling time of 40 h *in vitro* (Miller & Bell, 1987).

RPMI-8226 cells are passaged in suspension in RPMI-1640 supplemented with 15% foetal calf serum, 20 mM HEPES buffer, 100 IU $ml^{-1}$ penicillin, and 100 $\mu g\,ml^{-1}$ streptomycin. Initially an inoculum of $2\times10^7$ cells was injected s.c. and bilaterally into the flanks of 2 6–8 week-old male BALB/c athymic nude mice. After 12 days, tumours were palpable at each injection site. The growth of these tumours was monitored during the following 5 days (Figure 2a), after which time the animals were sacrificed by cervical dislocation and the tumours harvested. There was no evidence for invasion of tumour cells to the spleen, liver, lungs or retroperitoneal nodes.

One tumour was frozen in liquid $N_2$ immediately, a second was dissected, examined with a panel of monoclonal antibodies and established *in vitro*. The majority of the viable cells (>75%) were mouse 'T' lymphocytes. This was assessed using a fluorescein-conjugated rat anti-mouse monoclonal antibody directed against murine 'T' cell surface markers. (MAS 50, Sera-Lab. Crawley Down, Sussex). This population produced a pure culture within 7 days and had a doubling time of 11.8 h (Figure 2b). The cells all carried the MAS 50 'T' cell marker and had murine karyotype. Samples of the cells were examined by electron microscopy (Figure 1b). Unlike the human cell line, the mouse 'T' lymphoma (MIT 1) had a poorly developed endoplasmic reticulum and 'C' type virus particles could be seen budding from the plasmalemma. The murine origin of this lymphoma was confirmed by chromosome analysis and compared with the original human cell line. Figure 3a, b shows that the MIT 1 cells had telocentric chromosomes, characteristic of murine origin, whereas the RPMI-8226 cells do not have this type of chromosome. In human cells chromosomes are either meta, sub-meta or acrocentric.

The two remaining tumours, one from each animal were passaged bilaterally into the flanks of a further group of 16 athymic nude mice (see Figure 4). Large oedematous tumours, which were palpable on day 11, grew at each injection site. Animals were sacrificed before the tumours ulcerated, and samples of tumours were harvested and dissociated. Examination of these tumour cells with monoclonal antibodies and by karyotype showed that they were also murine 'T' lymphomas.

The growth of the tumour which had been stored in liquid $N_2$ was examined in homozygous and heterozygous immunocompetent BALB/c mice. The data summarized in Figure 4 show that tumours grew in immunocompetent animals. However it took 17 days for tumours to reach a mean diameter of 1 cm in these animals compared with 11 days in athymic nude mice. In each instance these tumours were murine 'T' lymphomas. When $5\times10^6$ MIT 1 cells were used an inoculum into mice of different phenotype, the time course of appearance of tumours was similar to that seen in animals which had received tumour cells from liquid $N_2$ (see Figure 4). Thus, each of the four tumours, which arose following the inoculation of the human cell line, RPMI-8226, into athymic nude mice, resulted from the growth of murine 'T' lymphomas.

In a second experiment, 4 female athymic nude mice were injected bilaterally in the flank with a bolus of $2\times10^7$ RPMI-8226 cells. Tumours appeared at two of the injection sites in separate animals 4 weeks after implantation. One of these tumours was a murine 'T' lymphoma, the second consisted of predominantly RPMI-8226 cells. The human myeloma cells were re-established *in vitro*. Examination of their karyotype showed that they are similar to the original cell line. Virus particles were not detectable using electron microscopy. No tumour growth occurred in the remaining two animals even though they received a further inoculation of $10^7$ RPMI-8226 cells 4 weeks after the original injection.

The observations that all these 'T' lymphomas were of mouse karyotype, produced tumours in immune-competent animals and contained 'C' type virus particles suggests that they resulted from the activation of murine leukaemia virus MuLV by the human myeloma cell line. Gautsch *et al.* (1980) reported the induction of MuLV by an oat cell carcinoma. However, no data were provided to show that the karyotype or isoenzyme patterns of the tumour remained human after passage *in vivo*. In other systems, the production of lymphomas in the lymph nodes and spleen of athymic nude mice has been ascribed to chronic antigenic stimulation producing a lymphoproliferative reaction (Baird

Correspondence: B.C. Millar.
Received 18 August 1987; and in revised form, 14 October 1987.

**Figure 1** Electron micrographs of (a) RPMI-8226 (human myeloma), arrow indicates typical well-developed endoplasmic reticulum characteristic of plasma cells ( x 33,000) and; (b) MIT 1 (mouse 'T' lymphoma) arrow indicates virus particle budding from the plasmalemma ( x 127,000).

**Figure 2** Growth of mouse 'T' lymphoma (a) in athymic nude mice and; (b) *in vitro* (MIT 1).

**Figure 3** Karyotype of (a) RPMI-8226 (human myeloma) ( x 8,500) and; (b) MIT 1 (mouse 'T' lymphoma) ( x 9,000).

*et al.*, 1982). Although there was no evidence for metastatic spread in the experiments reported herein this may have been due to the rapid growth of the primary tumours which necessitated the termination of experiments before the tumours ulcerated. Our findings concerning the rapid growth of these tumours are in agreement with those of Sparrow *et al.* (1986) who pointed out that since human tumour xenografts grow relatively slowly, rapid tumour growth and skin ulceration should alert the investigator of possible

**Figure 4** Flow diagram to show the attempted growth of RPMI-8226 cells (human myeloma) as xenografts in athymic nude mice.

malignant transformation of endogenous murine cells. The incidence of 'T' lymphomas in our system (5/6) is much higher than that reported by other workers. Sparrow *et al.* (1986) reported 3 murine fibrosarcomas which arose over a 6 year period from 30 human xenografts. In this respect the RPMI-8226 cell line appears more effective in initiating the production of endogenous virus which results in lymphoma production than they are of becoming established as a xenograft. The mechanisms of this virus induction remains obscure however, it is possible that RPMI-8226 cells produce a factor or hormone that acts as a potent inducer of endogenous 'C' type virus. A similar mechanism has been proposed to account for the induction of MuLV in nude mice bearing an oat cell carcinoma xenograft (Gautsch *et al.*, 1980).

Our results emphasize a serious pitfall that may be encountered when handling human tumour material as xenografts. Without examination of the resultant tumour cells *in vitro* for their growth pattern and against a panel of monoclonal antibodies directed against murine as well as human tissue any conclusion concerning the drug sensitivity of our myeloma cell line *in vivo* would have been meaningless. Furthermore, the data suggest that RPMI-8226 cells should be handled with caution since their ability to initiate the production of retrovirus may not be limited to viruses of murine origin.

### References

BAIRD, S.M., BEATTIE, G.M., LAMMON, R.A., LIPSICK, J.S., JENSEN, F.C. & KAPLAN, N.O. (1982). Induction of lymphoma in antigenically stimulated athymic mice. *Cancer Res.*, **42**, 198.

CUSTER, R.P., OUTZEN, H.C., EATON, G.J. & PREHN, R.T. (1973). Does the absence of immunological surveillance affect the tumour incidence in 'nude' mice? First recorded spontaneous lymphoma in a 'nude' mouse. *J. Natl Cancer Inst.*, **51**, 707.

GAUTSCH, J.W., KNOWLES, A.F., JENSEN, F.C. & KAPLAN, N.O. (1980). Highly efficient induction of type C retroviruses by a human tumour in athymic mice. *Proc. Natl Acad. Sci.* (USA), **77**, 2247.

HIRSH, N.S., PHILLIPS, S.M., SOLNIK, C., BLACK, P.H., SCHWARTZ, R.S. & CARPENTER, C.B. (1972). Activation of leukaemia viruses by graft-versus-host and mixed lymphocyte reactions *in vitro*. *Proc. Natl Acad. Sci.* (USA), **69**, 1069.

MATSUOKO, Y., MOORE, G.E., YAGI, Y. & PRESSMAN, D. (1967). Production of free light chains of immunoglobulin by a hematopoietic cell line derived from a patient with multiple myeloma. *Proc. Soc. Exp. Biol.*, **125**, 1246.

MERTENS, N., DEHOU, M.F., VANDERBRUGGEN, H., VAN RIET, I. & VAN CAMP, B. (1985). A monoclonal antibody (HAN-PCl) reacting with a maturation antigen on plasma cells. *Protides Biol. Fluids*, **32**, 891.

MILLAR, B.C. & BELL, J.B.G. (1987). Comparison of melphalan toxicity in human lymphocytic cells and Chinese hamster cells *in vitro*: The relationship between DNA-DNA cross-link formation and clonogenic survival. *Carcinogenesis*, **8**, in press.

PRICE, P.J., ARNSTEIN, P., SUK, W.A., VERNON, M.L. & HUEBNER, R.J. (1975). Type C RNA viruses of the NIH nude mouse. *J. Natl Cancer Inst.*, **55**, 1231.

SHARKEY, F.E., FOGH, J.M., HADJU, F.A., FITZGERALD, P.J. & FOGH, J. (1978). Experience in surgical pathology with human tumours grown in the nude mouse. In *The Nude Mouse in Experimental and Clinical Research*. Fogh, J. & Giovanella, B.C. (eds) 1, Academic Press: New York.

SPARROW, S., JONES, M., BILLINGTON, S. & STACE, B. (1986). The *in vivo* malignant transformation of mouse fibroblasts in the presence of human tumour xenografts. *Br. J. Cancer*, **53**, 793.

TODARO, G.J., ARNSTEIN, P., PARKS, W.P., LANNETTE, E.H. & HUEBNER, R.J. (1973). A type-C virus in human Rhabdoryosarcoma cells after inoculation into NIH Swiss mice treated with antithymocyte serum. *Proc. Natl Acad., Sci.* (USA), **70**, 859.


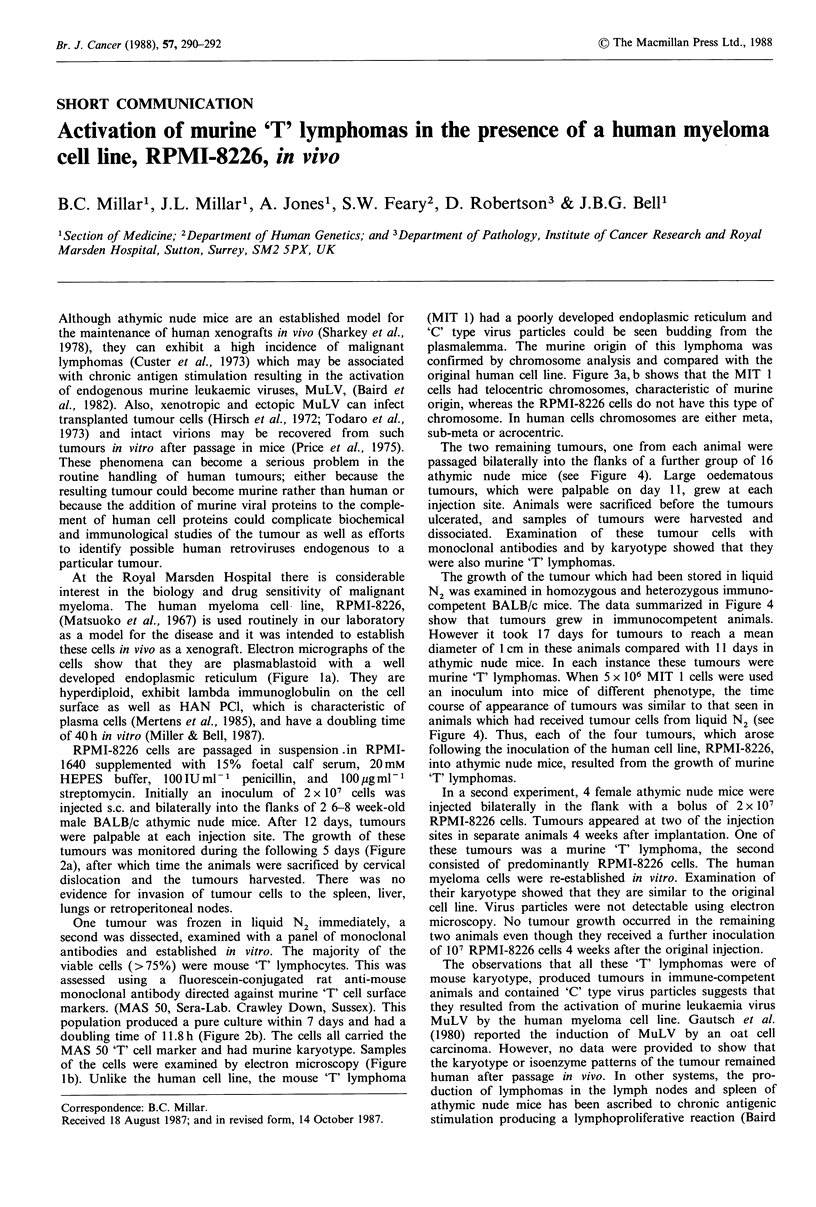

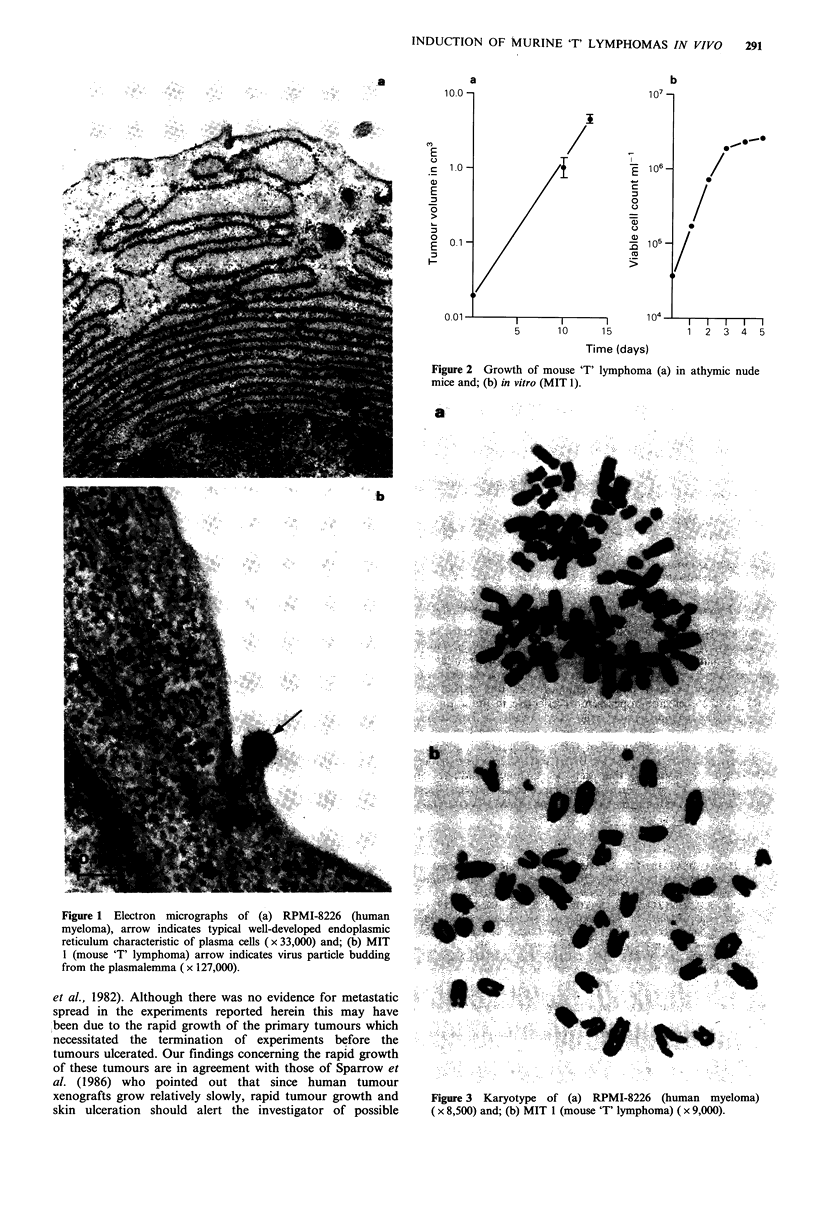

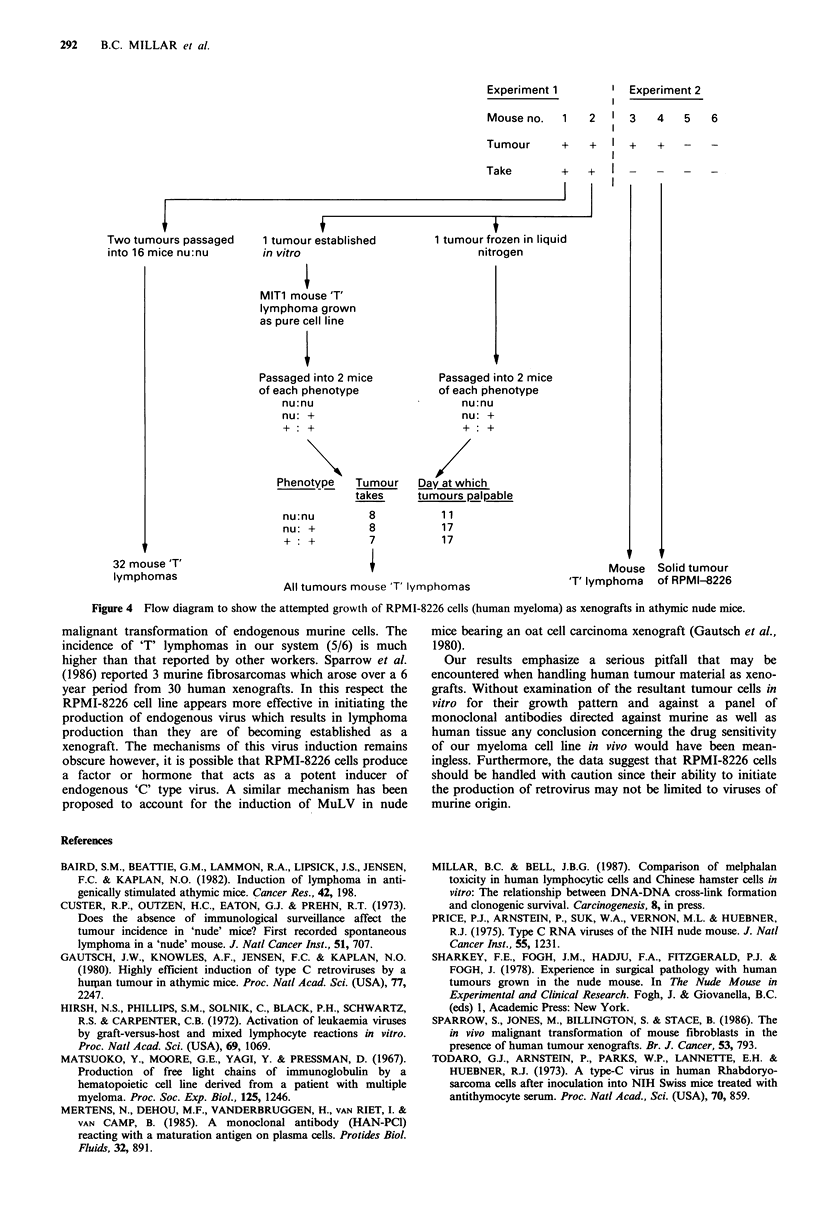


## References

[OCR_00284] Baird S. M., Beattie G. M., Lannom R. A., Lipsick J. S., Jensen F. C., Kaplan N. O. (1982). Induction of lymphoma in antigenically stimulated athymic mice.. Cancer Res.

[OCR_00289] Custer R. P., Outzen H. C., Eaton G. J., Prehn R. T. (1973). Does the absence of immunologic surveillance affect the tumor incidence in "nude" mice? First recorded spontaneous lymphoma in a "nude" mouse.. J Natl Cancer Inst.

[OCR_00295] Gautsch J. W., Knowles A. F., Jensen F. C., Kaplan N. O. (1980). Highly efficient induction of type C retroviruses by a human tumor in athymic mice.. Proc Natl Acad Sci U S A.

[OCR_00301] Hirsch M. S., Phillips S. M., Solnik C., Black P. H., Schwartz R. S., Carpenter C. B. (1972). Activation of leukemia viruses by graft-versus-host and mixed lymphocyte reactions in vitro.. Proc Natl Acad Sci U S A.

[OCR_00307] Matsuoka Y., Moore G. E., Yagi Y., Pressman D. (1967). Production of free light chains of immunoglobulin by a hematopoietic cell line derived from a patient with multiple myeloma.. Proc Soc Exp Biol Med.

[OCR_00325] Price P. J., Arnstein P., Suk W. A., Vernon M. L., Huebner R. J. (1975). Type-C RNA viruses of the NIH nude mouse.. J Natl Cancer Inst.

[OCR_00337] Sparrow S., Jones M., Billington S., Stace B. (1986). The in vivo malignant transformation of mouse fibroblasts in the presence of human tumour xenografts.. Br J Cancer.

[OCR_00342] Todaro G. J., Arnstein P., Parks W. P., Lennette E. H., Huebner R. J. (1973). A type-C virus in human rhabdomyosarcoma cells after inoculation into NIH Swiss mice treated with antithymocyte serum.. Proc Natl Acad Sci U S A.

